# Blockade of the Terminal Complement Cascade Using Ravulizumab in a Pediatric Patient With Anti-complement Factor H Autoantibody-Associated aHUS: A Case Report and Literature Review

**DOI:** 10.7759/cureus.19476

**Published:** 2021-11-11

**Authors:** Xiaoyan Wu, Amanda Szarzanowicz, Adinoyi Garba, Beverly Schaefer, Wayne R Waz

**Affiliations:** 1 Nephrology, University at Buffalo, Buffalo, USA; 2 Medicine, University at Buffalo Jacobs School of Medicine and Biomedical Sciences, Buffalo, USA; 3 D'Youville School of Pharmacy, University at Buffalo, Buffalo, USA; 4 Hematology and Medical Oncology, University at Buffalo Jacobs School of Medicine and Biomedical Sciences, Buffalo, USA; 5 Nephrology, University at Buffalo Jacobs School of Medicine and Biomedical Sciences, Buffalo, USA

**Keywords:** lactate dehydrogenase (ldh), hemolytic anemia, proteinuria, acute kidney injury, ravulizumab, eculizumab, complement c3, factor h autoantibody, factor h, atypical hemolytic uremic syndrome

## Abstract

Atypical hemolytic uremic syndrome (aHUS) is a rare disease in pediatrics with 6-10% of cases associated with complement factor H autoantibodies*. *Ravulizumab is a new treatment option available for long-term management through blockage of the terminal complement cascade. We report a case of a previously healthy eight-year-old female who presented with hemolytic anemia, thrombocytopenia, and acute kidney injury. Low complement C3, normal ADAMTS13, and negative rheumatology and infectious disease panels suggested aHUS. A follow-up complement aHUS/TMA gene panel was negative for ADAMTS13, C3, CD46, CFB, CFD, CFH, CFHR1, CFHR3, CFHR5, CRI, DGKE, PLG, and THBD mutations and positive for MCP/CD46 haplotype and CFH-H3 haplotype. Further testing found decreased factor H (B1H) plasma level and increased factor H autoantibody, suggesting anti-factor H antibody-associated aHUS. She received hemodialysis (2 treatments) and eculizumab was initiated promptly. The patient had complete renal recovery after one month of therapy, and anemia, thrombocytopenia, and hemolysis resolved after two months of therapy. After five months of therapy, eculizumab was successfully switched to ravulizumab. After 12 months of initial diagnosis, complement C3 and factor H normalized, however, factor H autoantibody remained elevated. The case supports the notion that timely recognition of anti-FH-associated aHUS is important for disease management and that early specific therapy with immunosuppression results in favorable outcomes. It also illustrates that the blockade of the terminal complement cascade using eculizumab holds promise for pediatric cases. Finally, eculizumab can be safely switched to ravulizumab with an optimal longer duration between treatments in the context of aHUS.

## Introduction

Hemolytic uremic syndrome (HUS) is characterized by hemolytic anemia, thrombocytopenia, and acute kidney injury. High lactate dehydrogenase (LDH), presence of schistocytes on a peripheral blood smear, and undetectable haptoglobin levels confirm intravascular hemolysis. Atypical HUS (aHUS) accounts for 5%-10% of all HUS cases in children. The atypical hemolytic uremic syndrome is a rare disease in pediatrics, with an annual incidence of 0.75 cases per million people 20 years of age and younger [1]. It can be distinguished from other thrombotic microangiopathies by ADAMTS13 activity greater than 10% ruling out thrombotic thrombocytopenic purpura (TTP) and by negative stool cultures and PCR for Shiga-toxin along with serology for anti-lipopolysaccharides ruling out Shiga-toxin-associated HUS. Primary aHUS is associated with dysregulation of the complement system. Low C3 plasma levels with normal C4 plasma levels are found in 30%-50% of aHUS patients [2]. Research has shown that mutations in regulatory proteins of the complement alternative pathways and in proteins of the C3 convertase have a role in the pathogenesis of aHUS. In 2005, it was first reported that anti-complement factor H autoantibodies (FH autoAb) can cause complement system dysregulation leading to aHUS through neutralization of the complement factor H regulatory protein [3]. FH autoAbs have since been found in 10.9% of aHUS patient cohorts in North America, consistent with reports from Europe, and in 55.8% of the patients in the aHUS database in India, with children aged four to seven most affected [4-5].

Prior to monoclonal antibody treatment, the prognosis of aHUS was poor with 55% of patients progressing to end-stage renal failure (ESRD) or death within three years of remission [6]. FH autoAb-associated aHUS was treated with plasma infusion, oral prednisone, oral mycophenolate, and IV rituximab previously [7]. Renal transplantation without a preventative strategy for antibody production was not found to be an effective long-term treatment plan [8]. Prognosis improved with the approval of eculizumab, a monoclonal antibody that is administered intravenously and binds specifically to C5, blocking its cleavage into C5a and C5b, effectively regulating the complement system cascade. It was the first therapeutic drug approved by the Food and Drug Administration (FDA) for the treatment of aHUS in pediatrics and adults in 2011. A new therapeutic drug, ravulizumab is the first and only long-acting C5 inhibitor that gained FDA approval in October 2019 for the treatment of aHUS. This therapy has the potential to improve quality of life and decrease cost by 34%-36% with reduced infusions and hospital visits, as dosing is every eight weeks in comparison to eculizumab, which is administered every two weeks.

## Case presentation

An eight-year-old, previously healthy girl presented to the emergency department (ED) with right upper quadrant abdominal pain, progressive nausea, and vomiting, and decreased urine output for five days. Her mother also noticed jaundice of the skin and sclerae. In the ED, vital signs were normal. Physical examination revealed mild tenderness to the right upper quadrant, pallor and jaundice of the skin, mild erythematous plaques on both cheeks, and scattered petechiae on the hands, thighs, lower legs, and feet as well as a few scattered ecchymotic areas on the lateral side of the upper left thigh.

Laboratory results revealed anemia (hemoglobin 5.1 g/dL; nl 11-14 g/dL), thrombocytopenia (platelets 39 x10^9/L; nl 150-450 x10^9/L), hyperbilirubinemia (bilirubin of 3.4 mg/dL and bilirubin direct 1.1 mg/dL), and acute kidney injury (serum creatinine 6.01 mg/dL; nl 0.4 mg/dL). High LDH (3070 unit/L; nl 120-235 unit/L), low haptoglobin (<8 mg/dL, nl 30-200 mg/dL), and presence of schistocytes on peripheral smears suggested hemolysis. Urine output was diminished and urinalysis was significant for proteinuria. Additionally, she had increased transaminase levels. An ultrasound of the abdomen found bilateral increased renal echogenicity with borderline increased size for age (right kidney measuring 10 cm in length and the left kidney measuring 9.6 cm in length), which could be seen in the setting of medical renal disease.

She was admitted to the pediatric intensive care unit (PICU) and started on conservative fluid management. She received pRBC transfusions and furosemide as supportive care with an improvement of her hemoglobin (11.9 g/dL), platelets (90 x 19^9/L), and LDH (1461 unit/L). She received intermittent hemodialysis twice on Days 2 and 3 of admission and serum creatinine (1.82 mg/dL) stabilized.

Rheumatology service was consulted and panels for systemic lupus erythematosus (SLE) and antineutrophil cytoplasmic antibodies (ANCA) vasculitis were negative. Infectious disease service was consulted and adenovirus, Streptococcal pneumoniae, and hepatitis were negative. Stool culture for Escherichia (E.) coli 0157:H7 was also negative. With low complement C3 (59 mg/dL), normal complement C4 (30.7 mg/dL), and normal ADAMTS13 (>100%), the diagnosis of aHUS following acute gastrointestinal (GI) illness without diarrhea was suspected.

Eculizumab was initiated promptly. She received meningococcal vaccines (conjugated and serotype B) with her first dose due to the risk of meningococcal disease with treatment, and she continued to receive penicillin V 250 mg BID for meningococcal prophylaxis. By Day 8, her renal function improved with a creatinine level of 1.05 mg/dL, her hematologic parameters stabilized, and she was discharged home.

**Figure 1:**
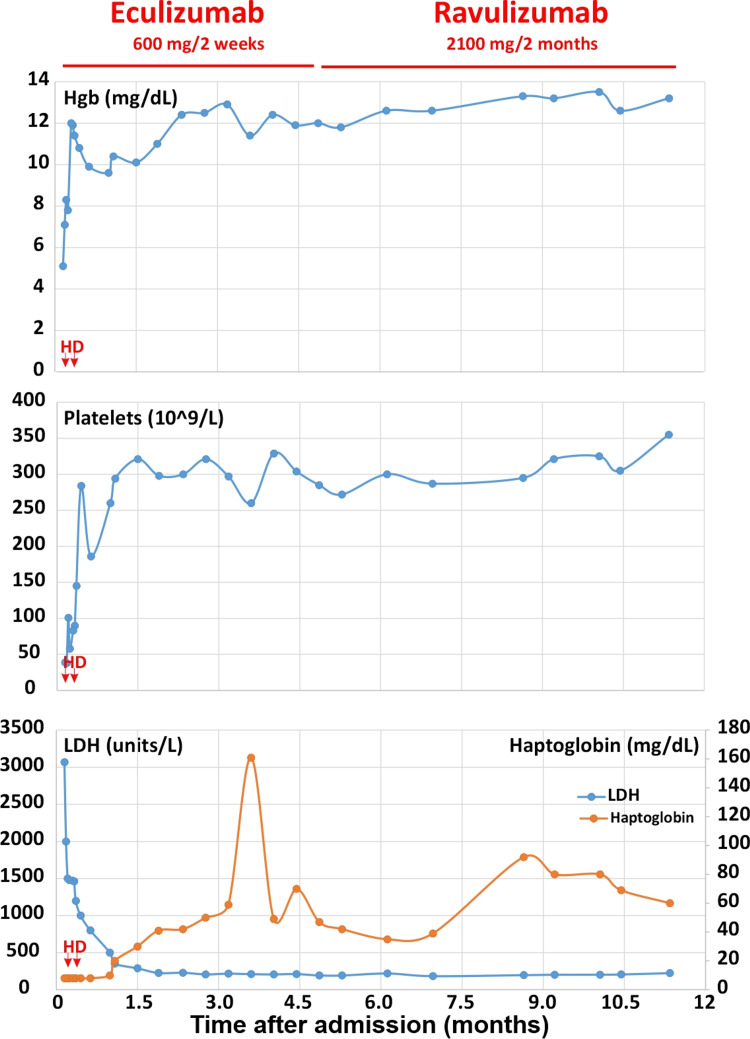
Responses of hemoglobin (Hgb), platelets, lactate dehydrogenase (LDH), and haptoglobin to anti-C5 antibody therapy Hemodialysis (HD) was initiated shortly after admission with two treatments during her hospital stay. Eculizumab was introduced after vaccination against meningococcal infection and continued for five months. The eculizumab infusion (every 2 weeks) was switched to ravulizumab infusion (every 2 months) successfully. As indicated, anemia, thrombocytopenia, and hemolysis resolved within two months of treatment with no recurrence after one year of therapy.

**Figure 2:**
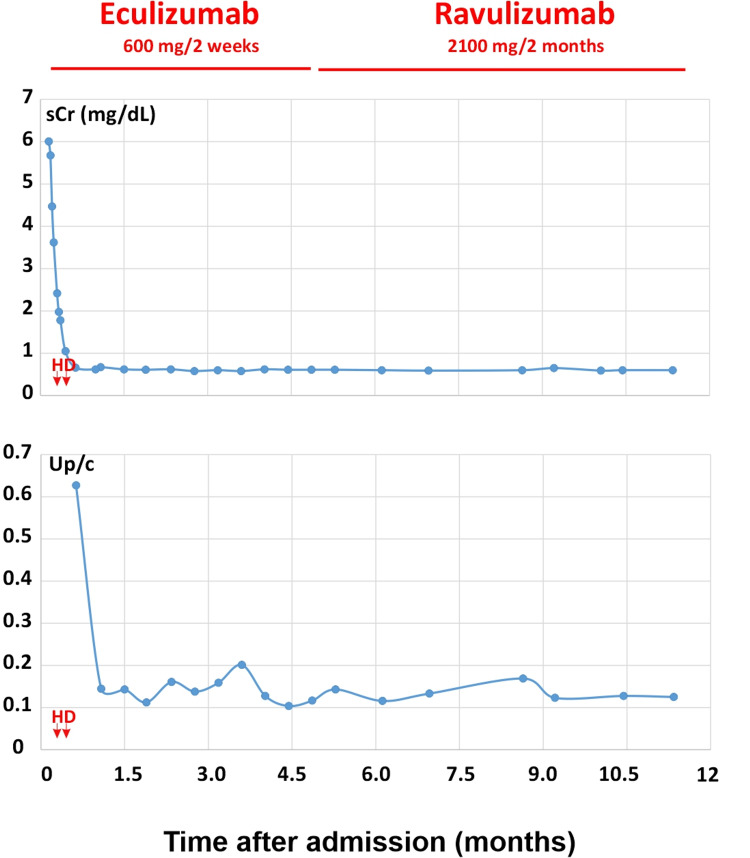
Responses of kidney function and proteinuria to anti-C5 antibody therapy Hemodialysis (HD) was initiated shortly after admission with two treatments during her stay. Eculizumab was introduced after vaccination against meningococcal infection and continued for five months. The eculizumab infusion (every 2 weeks) was switched to ravulizumab infusion (every 2 months) successfully. As indicated, acute kidney injury (serum creatinine (sCr) 6.01) and proteinuria (high urine protein/creatinine ratio) resolved within one month with treatment. The patient had complete renal recovery with sCr 0.5-0.6, estimated glomerular filtration rate (eGFR) > 95 ml/min/1.73 m^2. There is no recurrence after one year of anti-C5 antibody therapy.

**Figure 3:**
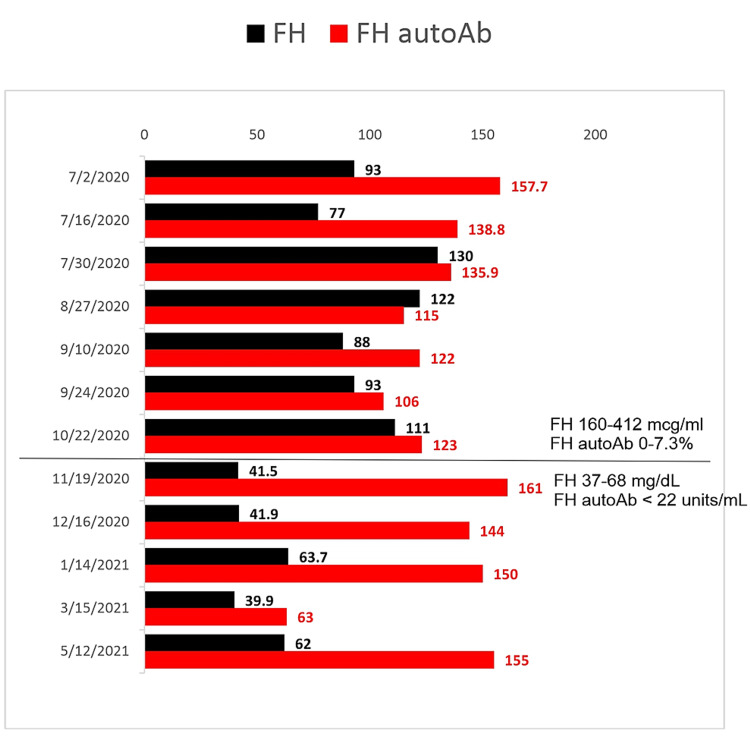
Levels of factor H (FH) and autoantibodies against factor H (FH autoAb) during anti-C5 antibody therapy The black line indicates test center change. Until October 22, 2020, FH and FH autoAb were analyzed at National Jewish Health (FH nl 160-412 mcg/ml, FH autoAb nl 0-7.3%). From November 19, 2020, they were analyzed at Cincinnati Children Hospital (FH nl 37-68 mg/dL, FH autoAb nl < 22 units/mL). Absolute values are indicated. After one year of therapy, FH returned to normal and FH autoAb remained elevated.

Atypical HUS-specific gene panel revealed no mutation for ADAMTS13, C3, CD46, CFB, CFD, CFH, CFHR1, CFHR3, CFHR5, CRI, DGKE, PLG, and THBD. She was positive for the MCP/CD46 and CFH-H3 haplotypes. Her complement factor H (FH) level was low at 77-93 mcg/ml (nl 160-412 mcg/ml, National Jewish Health) and complement factor H auto-antibody (FH autoAb) was high to 135-143% (nl 0-7.3%, National Jewish Health). She remained on eculizumab infusions 600 mg every two weeks. Her anemia, thrombocytopenia, and hemolysis resolved (Figure 1) within two months of therapy. She had complete renal recovery (Figure 2) within one month of initiation of eculizumab therapy. After five months of therapy, eculizumab was switched to ravulizumab to reduce frequent venous access and to reduce caregiver hospital visits. She received a loading dose infusion IV 900 mg and then received IV ravulizumab infusion IV 2100 mg two weeks later and every eight weeks thereafter. The kidney function remains within normal range with serum creatinine 0.5-0.6 mg/dL and eGFR > 95 ml/min/1.73 m^2^ during treatment. Complement C3 and FH normalized after 12 months of initial diagnosis. However, FH autoAb remains elevated (Figure 3). She continues on ravulizumab one year after initiating treatment with anti-C5 antibodies due to persistently elevated FH autoAb. She has tolerated therapy with no adverse side effects nor recurrence of aHUS.

## Discussion

HUS, or so-called diarrhea-positive HUS, is caused by exposure to Shiga toxin-producing E coli. Patients present with GI prodrome nausea, vomiting, abdominal pain, and diarrhea, which may progress to ileus, bowel ischemia, intussusception, and perforation. Renal involvement includes hematuria, proteinuria, and renal insufficiency severe enough to necessitate dialysis. Microangiopathic hemolytic anemia and extensive microvascular injury may result in childhood apraxia of speech, myocardial, pancreatic, and skeletal muscle injury. HUS without any accompanying diarrheal illness was previously classified as D-negative HUS, which is now recognized as a separate disease and is defined as atypical HUS (aHUS). It is estimated that aHUS accounts for 5%-10% of all cases of HUS in children. Primary aHUS is associated with dysregulation of the complement system with possible infectious triggers including seasonal predilection and prodromal symptoms such as fever, respiratory tract infection, or GI prodrome [5]. Secondary causes of aHUS include pregnancy, HIV, bone marrow and solid organ transplantation, and other malignancies [9].

**Figure 4:**
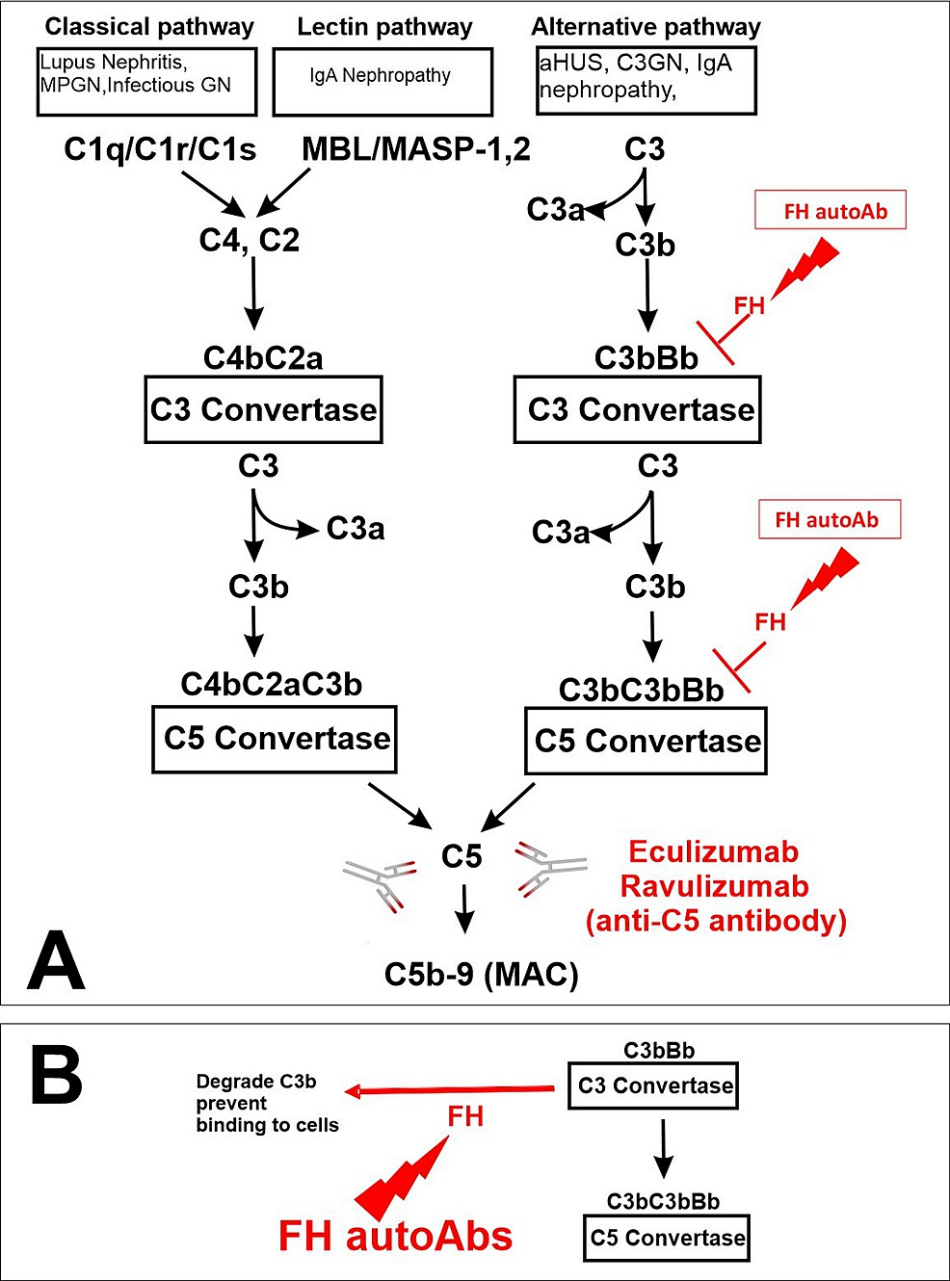
Alternative complement pathway in atypical hemolytic uremic syndrome (aHUS) A: FH regulates alternative pathways. Factor H autoantibodies (FH autoAb) prevent its catabolism, leading to alternative pathway (AP) dysregulation. This allows the alternative complement pathway to be “always on.” Ravulizumab (Ultomiris, Alexion Pharmaceuticals), a humanized monoclonal antibody, is a long-acting complement C5 inhibitor for the treatment of aHUS. Like the first-generation C5 inhibitor, eculizumab, ravulizumab binds specifically and with high affinity to the complement protein C5, thereby preventing the formation of the terminal complement complex C5b-9, which mediates cell lysis. Ravulizumab has been developed using Xencor’s antibody half-life prolongation technology, which utilizes antibody Fc variants to prolong half-life, allowing an extended dosing interval from two weeks to two months. B: Interaction between FH and FH autoAb.

The complement system is an innate defense mechanism against bacteria that functions through opsonization, phagocytosis, and lysis of pathogens. The complement system can be activated by the classical pathway, lectin pathway, or alternative pathway (Figure 4). aHUS is associated with overactivation or inadequate regulation of the alternative pathway. In the alternative pathway, complement C3 spontaneously hydrolyzes, resulting in the covalent attachment of C3b to bacterial cell surfaces. C3b can combine with factor B to form the C3 convertase. This enzyme complex has a positive feedback effect to amplify C3b and can combine with C3b to form the C5 convertase. Cleavage of C5 results in the anaphylatoxins C5a and C5b, the membrane attack complexes that initiate cell death [8].

Regulatory proteins protect host cells as C3b indiscriminately binds to host and pathogenic cells [10]. Complement factor I inactivates C3b with membrane cofactor proteins CD46 and FH. FH binds to glycosaminoglycans and heparin on host cells and to C3b to allow factor I to cleave C3b and additionally decays accelerating activity through the removal of factor B from the C3 convertase [8].

Mutations in four of the regulatory proteins have been identified to play a role in the pathogenesis of aHUS, including FH (identified in 24%-28% of aHUS patients), membrane cofactor protein (<5-9%), complement factor I (4%-8%), and thrombomodulin (0%-5%). Mutations in the two proteins of the C3 convertase, C3 (2-8%) and complement factor B (0-4%), also put carriers at a predisposition to aHUS. Additionally, the DGKE mutation, which causes permanent endothelial cell activation, has been found in 0%-3% of aHUS patients [11-12]. The absence of any known germline variants or autoantibodies is found in 30%-50% of patients with aHUS and the pathogenic mechanism for these patients has not been established [12]. Most recently, FH autoAbs of the IgM class were reported in 3.8% of patients suggesting additional pathogenesis for aHUS [13].

FH autoAbs of the IgG class were found in the patient we present and have been found in 6%-10% of all aHUS cases [14]. Antibodies form an immune complex with FH at the C-terminal, blocking FH from binding to glycosaminoglycans and heparin on host cells and C3b. FH autoAbs are strongly associated with complement factor H-related gene 1 (CFHR1) deficiency, with 80% of patients with FH autoAb-associated aHUS found to have homozygous CFHR1-CFHR4 deletion and 13.3% of patients to have heterozygous CFHR1 and CFHR3 deletion. In 6.7% of patients though, there are no genetic variants in CFHR genes as was in our patient [15]. The pathogenic mechanism is not well-defined and the presence of CFHR1 deficiency does not infer direct causation to FH autoAb-associated aHUS, as the CFHR1 allele frequency was found in 19.8% of healthy European Americans [16].

Haplotype CD46 and FH-H3 have been found to increase the penetrance of disease in aHUS patients with heterozygous likely pathogenic variants in genes that encode complement regulators and were identified in our patient. Interestingly, these haplotypes are not associated with increased risk in FH autoAb-associated aHUS [15].

Treatment and long-term management of FH autoAb-associated aHUS are important due to a high rate of relapse. In a multicenter study, relapse was found in 57% of FH autoAb-associated aHUS patients, with 68% of relapses occurring within six months of remission [14].

The prognosis of FH autoAb-associated aHUS was poor prior to monoclonal antibody treatment, with 63% of patients progressing to ESRD or death within three years of diagnosis [6]. Plasma therapy was the previously recommended first-line treatment for FH autoAb-associated aHUS. Additional long-term immunosuppressive treatment with steroids and azathioprine, mycophenolate mofetil, intravenous cyclophosphamide, or anti-CD20 was recommended to suppress FH autoAbs and reduce the risk of aHUS recurrence [10]. Treatment complications included morbidities associated with catheters in-situ that were seen in 31% of patients who had central venous catheter insertion and plasma hypersensitivity was seen in 16% of patients [17]. While renal transplantation has been shown to be effective in other types of aHUS, transplantation without a preventative strategy for antibody production was not found to be effective for FH autoAb-associated aHUS with a report of recurrence occurring in five of nine allografts transplanted [8].

Eculizumab (Solaris®, Alexion Pharmaceuticals, Cheshire, CT) was the first recombinant humanized monoclonal antibody approved for aHUS treatment by the US FDA, first for adult patients in May 2009 and later for pediatric patients in 2011. It was initially approved to treat paroxysmal nocturnal hemoglobinuria and is now additionally approved for the treatment of neuromyelitis optica. It inhibits the complement pathway by binding to complement protein C5, blocking the cleavage of C5 into C5a and C5b (Figure 4), therefore preventing the creation of membrane attack complexes and suppressing the complement system [10]. A prospective clinical trial found that after 26 weeks of eculizumab treatment, 64% of pediatric patients had a complete TMA response and the majority of participants had significant hematologic and renal improvement [18]. Of patients with aHUS, only 9% of children and 6-15% of adults progressed to end-stage renal disease or death within one year of follow up compared to 29% of children and 56% of adults who progressed to end-stage renal disease or death prior to eculizumab treatment [11]. Limitations to this treatment include financial costs, access to centers capable of delivering the drug, and bi-weekly intravenous administration in a clinical setting. Blockage of the complement system also has an increased risk of meningococcal infection and prophylaxis is required.

Ravulizumab (Ultomiris®, Alexion Pharmaceuticals, Cheshire, CT) was created by modifying eculizumab to extend the duration of the monoclonal antibody binding C5, resulting in an increase of the elimination half-life and lengthening of the dosing interval to every eight weeks [19]. The use of ravulizumab in pediatrics was approved in 2019. An initial study of the conversion of aHUS management from eculizumab to ravulizumab in pediatric patients found that ravulizumab was comparable to eculizumab in safety and efficacy [20]. Ravulizumab treatment-related costs were also found to be 34-36% lower than eculizumab treatment-related costs in pediatric patients [21].

Recommendations for the discontinuation of C5 inhibitors in aHUS patients in remission have not been well-defined. A recent prospective multicenter study found that the risk of aHUS relapse is increased in patients with complement gene variants within two years of eculizumab discontinuation and is safe in patients with no complement gene variants. Of the four patients with FH autoAb-associated aHUS in the study, 0 of the patients experienced relapse within two years of eculizumab discontinuation, which was initiated when antibody titer was decreased to 473-1,500 arbitrary units compared to 9,000-60,000 arbitrary units during active disease [22]. Antibody titers are found to be higher in patients one month prior to relapse compared to those who remain in remission [5]. Our patient continues on ravulizumab due to continued elevated FH autoAbs (Figure 3, 155 unit/ml, nl < 22 unit/ml) to prevent relapse.

## Conclusions

In conclusion, C5 inhibitors are an effective treatment for aHUS through the blockade of the terminal complement cascade. Ravulizumab is a cost-effective alternative to eculizumab that has the potential to increase the quality of life and reduce caregiver burden with extended dosing intervals. It can be safely switched from eculizumab in pediatric patients with FH autoAb-associated aHUS. Further study is necessary to determine the pathogenic mechanism of FH autoAb-associated aHUS and to standardize safe discontinuation of C5 inhibitor treatment.
